# Prognostic role of c-Met in head and neck squamous cell cancer tissues: a meta-analysis

**DOI:** 10.1038/s41598-018-28672-8

**Published:** 2018-07-10

**Authors:** Vit Vsiansky, Jaromir Gumulec, Martina Raudenska, Michal Masarik

**Affiliations:** 1 Department of Pathological Physiology, Faculty of Medicine, Masaryk University, Kamenice 5, 625 00 Brno, Czech Republic; 2 Department of Physiology, Faculty of Medicine, Masaryk University, Kamenice 5, 625 00 Brno, Czech Republic; 3 First Faculty of Medicine and BIOCEV, Charles University, Katerinska 32, 121 08 Prague 2, Czech Republic

## Abstract

This meta-analysis aims to evaluate the effects of high c-Met levels in head and neck squamous cell carcinomas (HNSCC) on survival and clinicopathological features. Publications concerned with the clinical significance of c-Met protein expression in HNSCC were identified from the Scopus and Web of Science database searches. To elucidate the relationship between c-Met expression and clinical outcomes, a meta-analysis of the selected articles was conducted. Seventeen publications involving a total of 1724 patients met the inclusion criteria. c-Met overexpression was significantly correlated with poor overall survival (hazard ratio (*HR*) = 2.19, 95% confidence interval (*CI*) = 1.55–3.10). c-Met immunohistochemical staining positivity was also associated with worse relapse-free survival (*HR* = 1.64, 95% *CI* = 1.24–2.17) and presence of regional lymph node metastases (odds ratio (*OR*) = 1.76, 95% *CI* = 1.26–2.45). High levels of c-Met expression in HNSCC predict unfavorable prognosis associated with common clinicopathological features.

## Introduction

c-Met is a transmembrane tyrosine kinase receptor for hepatocyte growth factor (HGF) also named scatter factor (SF). Its physiological function is connected to key processes in tissue embryogenesis and wound healing such as the ability of migratory cells to detach from the extracellular matrix, to elude anoikis and finally to settle in newly forming or damaged tissue^1^. By hijacking these pathways through c-Met activation, tumors increase their potential for invasive growth and metastasis^2^.

Head and neck squamous cell carcinoma (HNSCC) is the prominent histological type of head and neck cancer causing more than 200,000 deaths each year^3^. The currently most used prognostic system is the TNM classification which relies on gross pathological features of the tumor and has inherent limitations in its ability to predict the aggressiveness of the neoplasm^4^.

To address these issues, several biomarkers are currently under investigation as potential prognostic factors in HNSCC^5^. c-Met has recently gained significant attention due to it is being associated with the cancer stem cell population^6^ and also acting as a key component of resistance mechanism against epidermal growth factor receptor (EGFR) inhibition by novel therapeutics such as cetuximab^7^.

Owing to its importance in cancer progression, aberrant c-Met signalling has been studied specifically in the context of HNSCC mainly by identifying *MET* gene mutations and copy number alterations or by assessing c-Met overexpression^8^. While some studies have linked c-Met dysregulation to worse prognosis in HNSCC patients^9–11^, others have not found any significant correlation^12,13^. The aim of this meta-analysis is, therefore, to aid investigators and physicians in evaluating the role of c-Met as a robust prognostic factor in head and neck squamous cell carcinoma.

## Results

### Identification and characteristics of relevant studies

**Figure 1:**
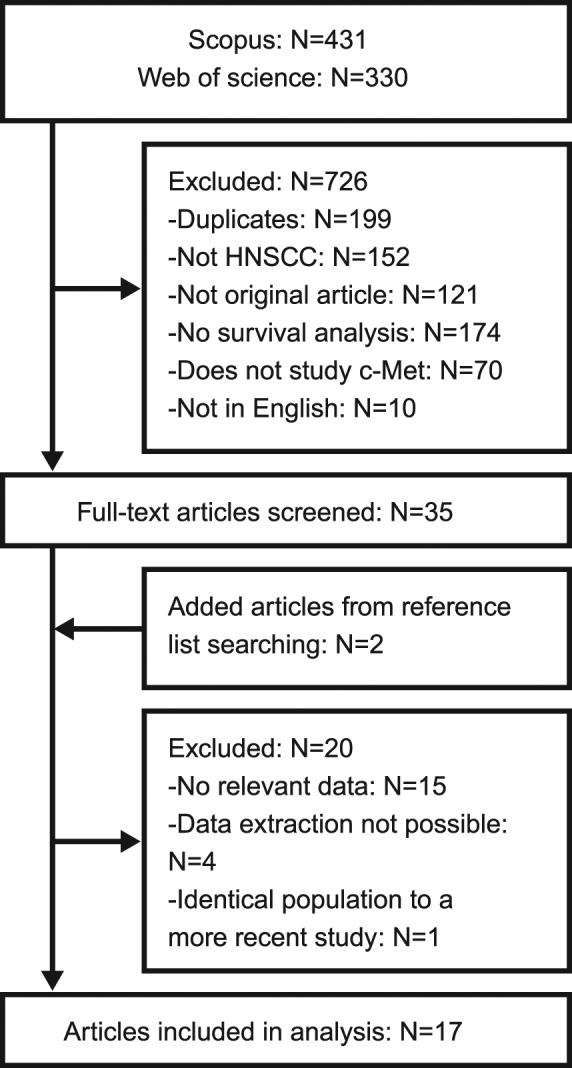
Flow chart of the article selection process.

The literature selection process of the eligible studies is presented in Fig. 1. The set of 17 final articles included 1724 patients with 101 tissue samples per study on average. The date of publication ranged from 2001 to 2017 with a median of the year 2011. A total of 8 studies were proceeded in Asia, 6 in Europe and 3 in the USA. All studies analyzed c-Met expression levels with immunohistochemical methods (IHC), which was chosen as the main and the only determinant of c-Met expression level in this meta-analysis. Several papers also investigated *MET* gene amplification status as a prognostic factor but the scarcity and heterogeneity of the data prevented a reasonable statistical analysis^11,12,14^. Only studies explicitly including squamous cell cancer were included. While 4 studies in total have explored c-Met expression levels in all various sites of the head and neck, 9 studies have focused only on the oral cavity, 3 only on oropharyngeal tumors and lastly, a single study included solely hypopharyngeal lesions. A subgroup analysis dissecting differences among these locations was not feasible due to lack of data on the association between clinical outcomes and the tumor site.

Fourteen studies explored the prognostic significance of c-Met in overall survival, while only 11 investigated relapse-free survival (i.e. disease-free survival). Association of several clinicopathological parameters with c-Met expression level was also studied by some of the selected papers. A single study only analyzed c-Met staining positivity and clinicopathological parameters but not patient survival^15^. Specifically, 12 articles evaluated N tumor staging, 10 also assessed T tumor staging and/or prognostic clinical staging, 9 studied tumor differentiation level (i.e. histological grading), 6 investigated the presence of distant metastases, 4 articles measured locoregional failure occurrences and finally, 2 papers provided p16 positivity values in high and low c-Met expression groups. Supplementary information presents all the studies included in the meta-analysis in detail.

### Relationship of c-Met expression with overall and relapse-free survival

**Figure 2:**
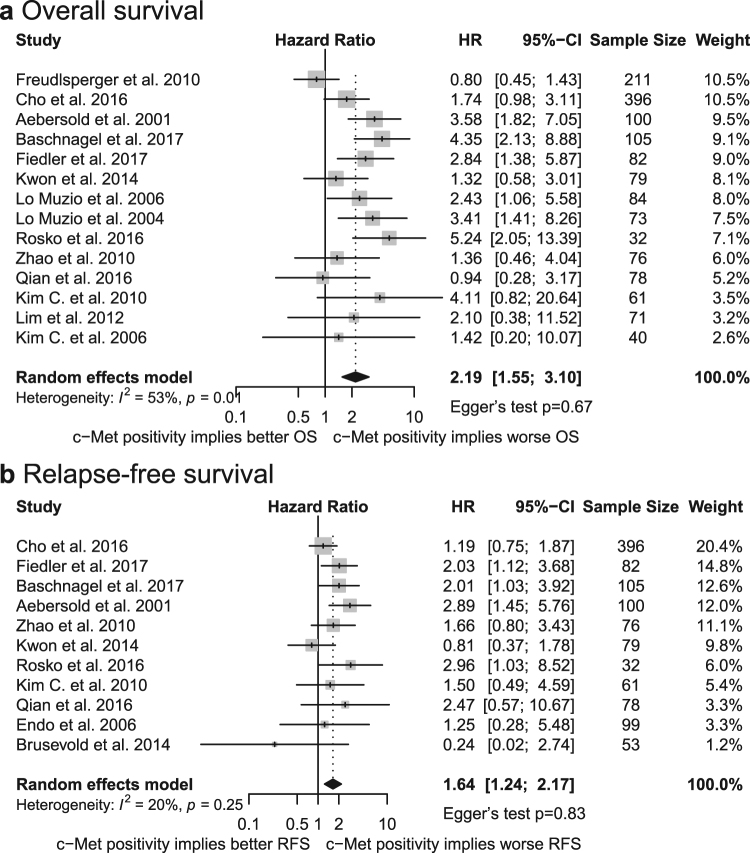
Forest plot of the association between c-Met staining positivity and OS (**a**) and between c-Met staining positivity and RFS (**b**). HR, hazard ratio; CI, confidence interval.

Meta-analysis of 14 applicable studies showed that positive staining for c-Met was associated with lower OS (*HR* = 2.19, 95% *CI* = 1.55–3.10; Fig. 2a)^9,10,12–14,16–24^. A moderate measure of heterogeneity was observed among these studies (*I*^2^ = 53%, *p*_*h*_ = 0.01). Combined data from a total of 11 studies also demonstrated a relationship between positive c-Met staining and poor RFS (*HR* = 1.64, 95% *CI* = 1.24–2.17; Fig. 2b) without significant interstudy heterogeneity (*I*^2^ = 20%, *p*_*h*_ = 0.25)^9,10,12,14,18,19,21–23,25,26^.

**Figure 3:**
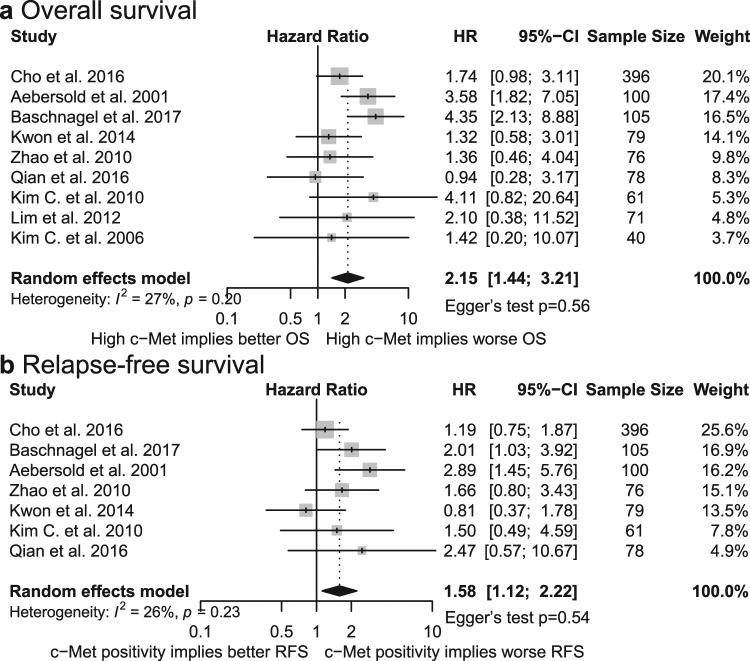
Forest plot of the association between c-Met expression and OS (**a**) and between c-Met expression and RFS (**b**) in studies with stricter cutoff criteria. HR, hazard ratio; CI, confidence interval.

Additional analysis of the 9 studies with stricter cutoff criteria revealed association of high c-Met expression and poorer OS (*HR* = 2.15, 95% *CI* = 1.44–3.21; Fig. 3a)^10,12,14,16,18,20–23^ without significant heterogeneity (*I*^2^ = 27%, *p*_*h*_ = 0.20). Seven of these studies also evaluated the impact of c-Met overexpression on worse RFS. Statistically significant association was found after combining the data (*HR* = 1.58, 95% *CI* = 1.12–2.22; Fig. 3b)^10,12,14,18,21–23^.

All studies were separated into groups based on the location where they were conducted (Asia vs. Europe and USA), sample size (equal to or greater than 100 vs. less than 100) and the ratio of c-Met positive to c-Met negative samples (equal to or greater than 50% vs. less than 50%). In all studied subgroups, c-Met staining positivity was associated with both poorer OS and RFS, except for the “Asian” subgroup which had a significant relationship only between c-Met staining positivity and worse OS but not between c-Met staining positivity and poorer RFS (Table 1).

**Table 1:** Subgroup analysis of the studies reporting the association of c-Met positivity and overall survival (OS) or relapse-free survival (RFS).

Factor	Subgroup	Sample characteristics		Survival statistics		Heterogeneity		Publication bias
		Studies N	Patients N	HR (95% CI)	p_hr_	I^2^ (%)	p_h_	Egger’s test p
**OS**								
	Overall	14	1488	2.19 (1.55–3.10)	<0.01	53	0.01	0.67
	Asia	6	723	1.67 (1.12–2.49)	0.01	0	0.88	0.54
	EU/USA	8	765	2.50 (1.54–4.07)	<0.01	70	<0.01	0.51
	Large Sample	4	812	2.12 (1.00–4.51)	0.05	83	<0.01	NA
	Small Sample	10	676	2.31 (1.63–3.28)	<0.01	6	0.39	0.60
	High c-Met positivity	8	721	1.73 (1.04–2.90)	0.04	51	0.05	0.65
	Low c-Met positivity	6	767	2.72 (1.80–4.13)	<0.01	44	0.11	0.35
**RFS**								
	Overall	11	1161	1.64 (1.24–2.17)	<0.01	20	0.25	0.83
	Asia	5	711	1.21 (0.88–1.68)	0.24	0	0.76	0.86
	EU/USA	6	450	2.22 (1.58–3.12)	<0.01	5	0.51	0.31
	Large Sample	3	601	1.81 (1.06–3.08)	0.03	59	0.09	NA
	Small Sample	8	560	1.57 (1.11–2.24)	0.01	8	0.37	0.45
	High c-Met positivity	5	368	1.98 (1.29–3.03)	<0.01	13	0.33	0.20
	Low c-Met positivity	6	793	1.52 (1.07–2.15)	0.02	26	0.24	0.7

HR, hazard ratio; CI, confidence interval; p_hr_, random effects model p values for hazard ratios; p_h_: test of heterogeneity p value; NA: not available.

### c-Met expression and clinicopathological features

c-Met staining positivity was significantly correlated with regional lymph nodes metastasis (*N0* vs. *N1* + *N2* + *N3*; *OR* = 1.86, 95% *CI* = 1.14–3.03; Table 2). No significant relationship was found between c-Met staining positivity and advanced T stage (*T1* + *T2* vs. *T3* + *T4*; *OR* = 1.26, 95% *CI* = 0.86–1.86), presence of distant metastasis (*OR* = 1.96, 95% *CI* = 0.88–4.37), locoregional failure (*OR* = 2.48, 95% *CI* = 0.97–6.35), poor tumor differentiation (*G1* + *G2* vs. *G3*; *OR* = 0.82, 95% *CI* = 0.46–1.43) or condensed TNM stage (*I* + *II* vs. *III* + *IV*, *OR* = 1.70, 95% *CI* = 0.90–3.19). Also, no correlation was found between high levels of c-Met and p16 positivity (*OR* = 0.65, 95% *CI* = 0.30–1.43), albeit only 2 studies investigated this parameter. Results of this analysis are presented in Table 2.

**Table 2:** association between c-Met staining positivity and clinicopathological features.

Clinicopathological feature	Studies N	Sample size	OR (95% CI)	I^2^ (%)	p_h_	Egger’s test p
T3/T4	10	1230	1.26 (0.86–1.86)	65	0.0.20	0.21
N^+^	12	1371	1.86 (1.14–3.03)	65	0.01	0.22
Distant metastasis	6	675	1.96 (0.88–4.37)	6	0.38	0.57
Stage III/IV	10	883	1.70 (0.90–3.19)	65	<0.01	0.69
Poor differentiation	9	679	0.82 (0.46–1.43)	42	0.09	0.43
Locoregional failure	4	282	2.48 (0.97–6.35)	59	0.06	NA
p16^+^	2	475	0.65 (0.30–1.43)	58	0.12	NA

OR: odds ratio; ph: test of heterogeneity p value; NA: not available.

### Publication bias

Publication bias was analyzed for OS, RFS and all subgroup analyses larger than 5 studies. Upon visual inspection of Begg’s funnel plots, no obvious asymmetry was noted (Supplementary information). Further investigation by Egger’s test also did not provide evidence for publication bias in any of the aforementioned parameters (Table 1, Figs 2 and 3).

## Discussion

Identification of prognostic markers enables stratification of patients into high-risk groups for whom a specific therapy could be necessary. In this meta-analysis, a significant relationship between high c-Met expression level and poor overall survival in the context of head and neck squamous cell carcinoma has been demonstrated.

Even though immunohistochemistry was used in all the included studies, a primary obstacle in interpreting and combining the outcome data was the diversity of cutoff definitions for the high and low expression patient groups used by the authors. In some studies, the chosen criteria have taken into account all the possible immunohistochemical factors and at the same time have been strict enough to warrant the usage of the term high/low c-Met expression. In other included articles, the criteria have not been clear or stringent enough and thus the separated patient groups can only be recognized as c-Met staining positive or negative, while the c-Met staining positive group could have expression levels ranging from fairly low, through moderate to high. To improve the interpretability of the results, overall and relapse-free survival values were calculated for all of the studies taken together and then separately for the high/low expression studies with stricter criteria. With all the studies combined, c-Met staining positivity was associated with poorer overall and relapse-free survival. When considering only the subset of studies with stricter cutoff criteria, high c-Met expression was correlated with worse overall survival but not with poorer relapse-free survival. This discrepancy could have arisen due to reduced statistical power when analyzing only a subset of all the possible studies. A unified method of scoring tissue samples for c-Met overexpression would greatly improve the comparability of future studies. A possible candidate for this role is a scoring method used in three of the eight high/low expression studies, the only one employed by more than a single group of authors.

Subgroup analysis showed that neither sample size, c-Met positive to c-Met negative ratio, nor the stratification of patients by Asian and Western countries have influenced the statistical significance of the association of c-Met staining positivity with poor overall survival. This was also true for the relationship between c-Met staining positivity and relapse-free survival, except for the Asian subgroup, where an insignificant hazard ratio was calculated, perhaps due to a higher measure of survival data extraction from Kaplan-Meier curves and therefore introduced error in the Asian subgroup.

The biological role of c-Met provides a possible explanation for its association with poor prognosis. Aberrant HGF/c-Met signalling has been previously shown to suppress E-cadherin expression and on the other hand, increase expression of matrix metalloproteinases thus inducing a migratory phenotype in the affected cells^27,28^. Indeed, in the present meta-analysis, a relationship between c-Met positivity and regional lymph node metastases was observed. Furthermore, a previously published study has shown c-Met-positive head and neck cancer cells to possess cancer stem cell properties and to contribute to chemo- and radiotherapy resistance^6^. HPV positivity of head and neck tumors is usually accompanied by p16 positivity and can have very different outcomes when compared to HPV negative cancers^29^. A relationship between c-Met positivity and p16 positivity could be in part responsible for these varying outcomes. However, no statistically significant correlation was found in our study.

There are several limitations which must be kept in mind when interpreting the results of this meta-analysis. First, usage of different antibodies against c-Met among studies contributes to ambiguity. Second, the number of patients in each study is generally small, thus reducing the power and precision of subgroup analyses. Lastly, only manuscripts published in English were considered for inclusion in this study, ignoring potential high-quality articles in other languages.

In summary, this meta-analysis shows that c-Met overexpression is an indicator of poor overall survival in head and neck squamous cancer patients, while c-Met immunohistochemical staining positivity is associated with worse overall and relapse-free survival as well as higher rates of regional lymph node metastases. These findings can help expand the application of c-Met as a prognostic marker in the clinical setting.

## Methods

### Search strategy and selection criteria

A systematic search of the published literature was conducted in the Scopus and Web of Science databases using the following terms: (c-met OR “kinase MET” OR “MET kinase” OR HGFR OR SFR OR “scatter factor receptor” OR “hepatocyte growth factor receptor”) AND (hnscc OR (“head and neck” AND cancer) OR ((*phary* OR ling* OR oral OR laryn*) AND (cancer OR tumo* OR carcinoma*))). Titles and abstracts of the resulting studies were reviewed to remove unrelated papers. The remaining part of the studies was read in full with the reference lists scanned for relevant articles.

In order to be selected, a study had to meet the following inclusion criteria: (1) was published in English; (2) measures c-Met protein level in human head and neck tumors; (3) provides survival data such as hazard ratio (HR) of overall survival (OS) or relapse-free survival (RFS) or corresponding Kaplan-Meier curves or provides data correlating c-Met protein level and clinicopathological features such as tumor TNM staging. When multiple articles included the same population of patients, only the most recently published paper was considered. The eligibility of the studies for meta-analysis was evaluated by two authors (V.V. and J.G.).

### Data extraction

The following characteristics were extracted from each eligible study: (1) age and sex of the patient population; (2) follow-up length; (3) number of cases in c-Met positive and negative groups; (4) TNM staging, clinical staging, histological grades and anatomical site of the tumor samples; (5) c-Met positivity definition and cutoff values; (6) HRs with 95% CIs for OS and RFS or corresponding Kaplan-Meier curves. Preferentially, values calculated with multivariate (i.e. multivariable) Cox proportional hazard model were used in the meta-analysis. If these were not available, results from univariate Cox regression or estimates from Kaplan-Meier survival curves were utilized instead. Calculation of HR from Kaplan-Meier curves was conducted according to Tierney *et al*.^30^. Extracted data are available at Supplementary dataset 1.

### Cutoff definitions for high c-Met expression

Due to no clear and standardized criteria for stratification of patients into high- and low-expression groups based on c-Met IHC results, authors of the included studies employed a wide variety of scoring algorithms to distinguish these two patient groups. Of the 17 included studies, only 9 have evaluated both staining intensity along with the relative number of cells stained and also had stringent enough cutoff criteria to truly separate patients into high- and low-expression groups^10,12,14,16,18,20–23^. Of these 9 articles, three have used a completely identical cutoff definition^16,21,31^. The 8 remaining papers have based their cutoff values only on either weak staining intensity or a relatively small proportion of stained cells without explicitly mentioning any consideration was given to the strength of the staining^9,13,15,17,19,24–26^.

Based on this, the 9 selected studies with stricter cutoff criteria have also been analyzed separately as c-Met high expression studies while all articles taken together are further termed c-Met positivity studies (Supplementary information).

### Statistical analysis

The expression of c-Met was determined as high or low, according to the definition and cutoff values of each study. To evaluate the effect of association between c-Met expression and OS or RFS, the hazard ratio of patients with low c-Met expression vs. patients with high c-Met expression was used. HR > 1 implied worse prognosis for the patients with high levels of c-Met expression.

All analyses were carried out using the R packages meta (version 4.8–4, https://CRAN.R-project.org/package = meta) and metafor (version 2.0-0, https://CRAN.R-project.org/package = metafor). Because each included study was done on a different population and using varying c-Met expression cutoff values, a random effects model was used to combine the data. Heterogeneity among studies was evaluated by the restricted maximum-likelihood (REML) method. Publication bias was first assessed graphically by Begg’s funnel plot analysis and consequently using Egger’s test with significant publication bias defined as p < 0.05. Only parameters with at least 5 applicable studies were analyzed for publication bias.

## Electronic supplementary material

Supplementary information

Supplementary dataset
