# Supplementary material for: Prognostic role of c-Met in head and neck squamous cell cancer tissues: a meta-analysis
Source: Sci Rep. 2018 Jul 10;8:10370. doi: 10.1038/s41598-018-28672-8 (PMC6039483; doi:10.1038/s41598-018-28672-8)

| Supplementary data for article |
| --- |
|  |
| Prognostic role of c-Met in head and neck squamous cell cancer tissues: a meta-analysis |
|  |
| Vit Vsiansky^1^, Jaromir Gumulec^1,2*^, Martina Raudenska^2^, Michal Masarik^1,2^ |
|  |
| ^1^Department of Physiology, Faculty of Medicine, Masaryk University, Kamenice 5, 625 00 Brno, Czech Republic |
| ^2^Department of Pathophysiology, Faculty of Medicine, Masaryk University, Kamenice 5, 625 00 Brno, Czech Republic |
|  |
| Corresponding author |
| *Dr. Jaromir Gumulec, Department of Pathological Physiology, Faculty of Medicine, Masaryk University, Kamenice 5, CZ-625 00 Brno, Czech Republic; e-mail: j.gumulec@med.muni.cz Phone: +420-5-4949-8526 |

# Main characteristics of included studies

| Author year | Country | Sample size | Follow-up median/mean* (months) | Outcome | Hazard ratio data |
| --- | --- | --- | --- | --- | --- |
| Baschnagel 2017 | USA | 105 | 35 | OS RFS | R |
| Fiedler 2017 | Germany | 82 | 17.4* | OS RFS | R |
| Cho 2016 | Korea | 396 | 37.1 | OS RFS | R |
| Rosko 2016 | USA | 32 | NA | OS RFS | R |
| Kwon 2014 | Korea | 79 | NA | OS RFS | E |
| Brusevold 2014 | Norway | 53 | 39 | RFS | E |
| Lim 2012 | Korea | 71 | 73.2 | OS | E |
| Freudlsperger 2010 | Germany | 211 | 42 | OS | E |
| Kim C. 2010 | Korea | 61 | NA | OS RFS | R |
| Endo 2006 | Japan | 99 | NA | RFS | R |
| Lo Muzio 2006 | Italy | 84 | NA | OS | R |
| Kim C. 2006 | Korea | 40 | NA | OS | E |
| Aebersold 2001 | Switzerland | 100 | 31.2 | OS RFS | R |
| Qian 2016 | USA | 78 | NA | OS RFS | R |
| Zhao 2011 | China | 76 | 41 | OS RFS | E |
| Lo Muzio 2004 | Italy | 73 | NA | OS | E |
| Klosek 2004 | Japan | 84 | NA | NA | NA |

# Definition of Cutoff values for individual studies

| Study | Cutoff definition |
| --- | --- |
| **Expression studies** |  |
| Baschnagel 2017 | Vast majority of specimens demonstrated diffuse (>50%) staining, therefore, patients were divided into low c-Met expression (+ or ++) or high c-Met expression (+++) based only on staining intensity. + = weak; ++ = intermediate; +++ = intense |
| Cho 2016 | Multiplication of staining intensity (0 = no staining; 1 = weak or barely detectable staining; 2 = distinct brown staining; 3 = strong dark brown staining) by the percentage (0-100%) of stained cells yields a score in the range of 0 and 300. A receiver operating characteristic curve analysis based on the MET copy number gain was then conducted to determine the c-Met expression cutoff value at score = 65, with the median score being 60. |
| Kwon 2014 | Staining intensity (0 = none; 1 = weak; 2 = moderate; 3 = strong) and the proportion of positively stained cells (0 = none; 1 = 0%-10%; 2 = 11%-30%; 3 = 31%-75%; 4 = >75%) were summed up to calculate a score in the range of 0-7. Patients with a score of at least 4 were considered as having high expression. |
| Lim 2012 | Samples were evaluated for staining intensity as follows: - no staining, ± definite but weak staining, + moderate staining, and ++ strong staining. Patients with at least + staining intensity and with more than 30% of the cells stained were considered as having high c-Met expression. (Same as Kim C. 2010 and Kim C. 2006) |
| Kim C. 2010 | Samples were evaluated for staining intensity as follows: - no staining, ± definite but weak staining, + moderate staining, and ++ strong staining. Patients with at least + staining intensity and with more than 30% of the cells stained were considered as having high c-Met expression. (Same as Lim 2012 and Kim C. 2006) |
| Kim C. 2006 | Samples were evaluated for staining intensity as follows: - no staining, ± definite but weak staining, + moderate staining, and ++ strong staining. Patients with at least + staining intensity and with more than 30% of the cells stained were considered as having high c-Met expression. (Same as Lim 2012 and Kim C. 2010) |
| Aebersold 2001 | Both staining intensity and the relative number of stained cells were considered. Patients with at least ++ (moderate) immunoreactivity were considered as having high c-Met expression. |
| Qian 2016 | Score as Weighted Index (WI). WI = intensity x % of positive staining. Intensity of staining scored as negative (0) weak (1), intermediate (2), and strong (3). Samples with WI of at least 70.416 (median value) were consider c-Met high expression. |
| Zhao 2010 | Samples with at least 30% of cells stained moderately (+) or strongly (++) were considered high-expression. |
| **Only staining positivity studies** |  |
| Fiedler 2017 | The median percentage of stained cells was 80%, the expression was therefore considered high if the percentage of stained tumor cells was higher than 80%. |
| Rosko 2016 | Staining intensity of 0 was considered negative while intensities 1 to 3 were considered positive. |
| Brusevold 2014 | Staining of at least 50% of cells was used as the cutoff criterion. |
| Freudlsperger 2010 | C-Met positivity was defined as at least 50% cells stained as determined by an image analysis software |
| Endo 2006 | Samples with at least 45% stained cells were considered c-Met positive. |
| Lo Muzio 2006 | Samples with at least 30% stained cells were considered c-Met positive. |
| Lo Muzio 2004 | Samples with at least 50% stained cells were considered c-Met positive. |
| Klosek 2004 | Staining intensity of 0 and 1 was considered negative while intensities 2 and 3 were considered positive |

# Begg’s plots for overall and relapse free survival for all studies (first row) and studies with stricter criteria (bottom row)


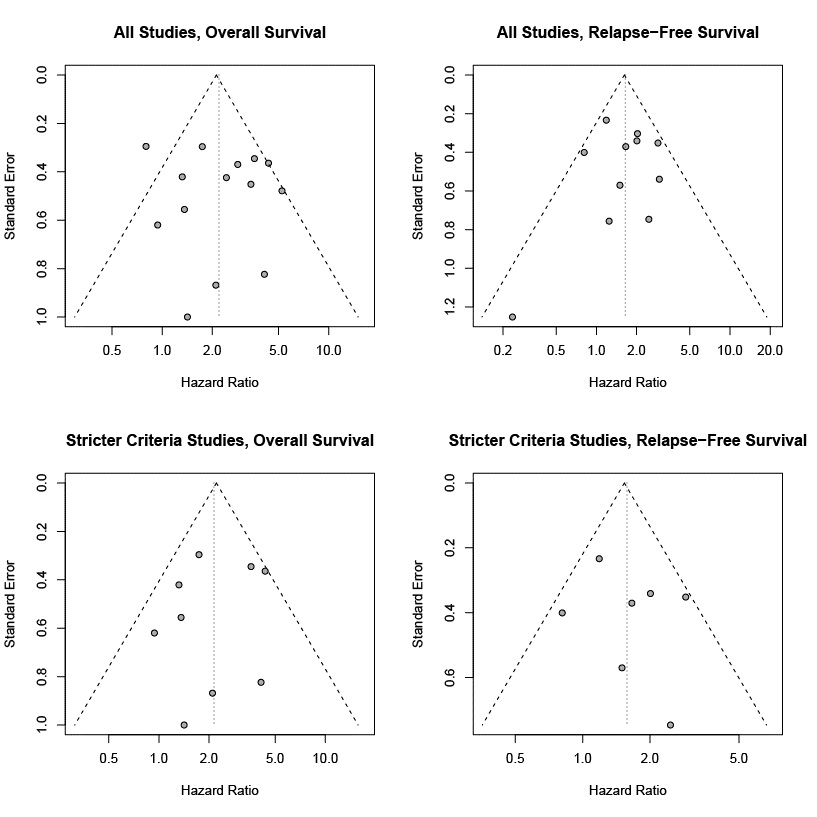

Supplement: Supplementary file 1 — Supplementary information [file 41598_2018_28672_MOESM1_ESM.docx]
